# Investigating the Relationship between the Dimensions and Morphology of Sella Turcica with the Long-Face Growth Pattern and the Vertical Growth Pattern

**DOI:** 10.1155/2023/9414184

**Published:** 2023-08-19

**Authors:** Ali Nabavizadeh, Negar Zeini, Ali Azarm, Parvin Khalili, Fatemeh Hajipour, Sarah Khaghani

**Affiliations:** ^1^Department of Orthodontics, School of Dentistry, Rafsanjan University of Medical Sciences, Rafsanjan, Kerman, Iran; ^2^Department of Oral and Maxillofacial Radiology, School of Dentistry, Rafsanjan University of Medical Sciences, Rafsanjan, Kerman, Iran; ^3^Student Research Committee, Rafsanjan University of Medical Sciences, Rafsanjan, Iran; ^4^Department of Epidemiology, School of Public Health, Social Determinants of Health Research Centre, Rafsanjan University of Medical Sciences, Rafsanjan, Iran

## Abstract

**Introduction:**

The sella turcica is one of the important landmarks of lateral cephalometry, which is used in orthodontics for the diagnosis, treatment plan, and evaluation of skeletal development and maturity. The purpose of the present study is to investigate the relationship between the dimensions and morphology of sella turcica with the long-face growth pattern and people with an open bite. This study also examines the relationship between sella turcica bridging (STB) and the vertical growth pattern.

**Methods:**

As many as 153 radiographs were analyzed using the Romexis software, considering the basal, gonial, and FMA angles to determine the vertical growth pattern of the face. The basal angle was also used to check for an open bite. Of these patients, 80 had a long vertical face growth pattern, and 73 had a normal face growth pattern. The four landmarks of tuberculum sellae, dorsum sellae, sellae floor, and posterior clinoid were determined on the cephalograms to measure the length, depth, and anteroposterior diameter of the sella turcica.

**Results:**

In this study, it was found that the chance of developing a long face in people with partial and complete bridging is 8.37 and 1.92, respectively. An increase in the length of the sella turcica for one unit decreases the chance of a long face, and as the depth of the sella turcica increases, the chance of a long face increases.

**Conclusions:**

STB is frequently seen in people with long faces. However, this finding should be considered in relation to other diagnostic parameters. The shorter the length and higher the depth of sella turcica, the higher the chance of developing a long face.

## 1. Introduction

The primary purpose of cephalometry is to research the growth pattern of the skull and face complex. Lateral cephalometry is the most common radiography in orthodontics for the diagnosis, treatment plan, and evaluation of skeletal development and maturity [1]. Cephalometric analysis uses landmarks to evaluate the position of the maxilla and mandible and their relationship with each other and the skull. These landmarks measure the location and position of specific structures, such as the maxilla or mandible, in relation to the cranium or themselves. The sella point (S), located in the sphenoid bone's sella cavity, is a common landmark used in cephalometric tracing [2].

According to studies conducted in this area, ossification in the tuberculum of the sella and bone resorption in the posterior parts of the sella reach their peak between the ages of 16 and 18 years [3, 4]. The morphology of sella turcica is formed at the beginning of embryonic development, and there will be almost no significant changes after the age of 12 years. The morphology of sella turcica is important in evaluating the morphology of the skull and late developmental changes [5, 6].

The development of brain tissue is closely related to the surrounding bone, and analyzing the bone can identify any congenital malformations in brain development. Therefore, abnormal shapes of the cranium base and sella turcica should be considered when evaluating craniofacial malformations [7]. Any disorder or disease in the pituitary gland can be manifested as a change in the shape or size of the sella turcica. Therefore, in addition to orthodontic treatments, it is important to evaluate the lateral cephalometric radiography of the patient in order to examine the abnormal and pathological conditions of the pituitary gland [8].

An increase in the size of the sella turcica in lateral cephalometric radiography can indicate diseases that cause an increase in pituitary hormone secretion. Additionally, radiographic images may show an enlarged sella turcica in cases of tumors such as pituitary macroadenoma, craniopharyngioma, intrasellar aneurysm, and meningioma. Conversely, primary pituitary insufficiency, Williams syndrome, and pituitary gland necrosis can cause a reduction in the dimensions of the sella turcica. Changes in the shape of the sella turcica may also be observed in connection with certain genetic disorders, including Down syndrome, Williams syndrome, lumbosacral myelomeningocele syndrome, and velocardiofacial syndrome [8–15].

Clinicians need to be familiar with the normal anatomy and diverse morphology of the sella turcica to detect any changes in shape or size that may indicate pathological conditions before they become clinically apparent [7]. Sella bridging is seen more frequently in patients with craniofacial anomalies such as Down syndrome and Williams syndrome [16, 17], as well as people with Class 3 growth patterns, dental anomalies and displacement, and latent canine [3, 18–20].

Patterns of facial growth in different people are normally horizontal and vertical [21]. Long face is an unbalanced face pattern in which the lower height of the front of the face (anterior nasal spine to menton) increases compare to the upper height of the front of the face (nasion to anterior nasal spine) [22]. The analysis of lateral cephalometric findings is essential in diagnosing the growth pattern in the vertical dimension [23].

Cephalometric analysis of patients with the long face deformity shows an increase in the angle between the mandibular plane and the occlusal plane (Schudy) [24]. These people usually have an anterior open bite due to the increase in height of the lower third of the face [25]. Due to its wide and multifactorial etiology and the possibility of returning to the initial state after treatment, the anterior open bite is considered one of the most challenging orthodontic problems to treat [26, 27]. The prevalence of open bites is different for different ages and ethnicities [28, 29]. Open bites can be classified into two types: dental and skeletal. A dental open bite is a malocclusion where there is no vertical overlap between the incisal edges when the posterior teeth are in contact. Skeletal open bite is determined based on the basal angle, and if the angle is greater than 27°, it is classified as skeletal open bite [30]. Several factors contribute to the etiology of skeletal open bites, including sucking habits, large lymphatic tissues and tonsils, an inappropriate skeletal growth pattern, incorrect position of the tongue and lips, a narrow maxilla, and hereditary factors [29, 31, 32].

To ensure proper treatment planning for open bite patients, it is crucial to accurately diagnose the underlying factors causing the condition. The treatment mechanism varies depending on the diagnosis, making it essential to identify the cause accurately. By establishing a relationship between the morphological changes of sella turcica and the type and severity of malocclusion, it can serve as a diagnostic sign in childhood to predict the quality and severity of malocclusion in adulthood. This approach can help to prevent the waste of time and money and loss of enthusiasm and motivation for both the patient and dentist during treatment. So the aim of this study was to investigate the relationship between the dimensions and morphology of sella turcica with the long-face growth pattern and the vertical growth pattern.

## 2. Materials and Methods

This case–control study was performed on patients of a specialized orthodontic clinic in Rafsanjan. Patients who were previously diagnosed with normal or long-face growth pattern from January 2021 to January 2022 entered in this study. The inclusion criteria were high-quality lateral cephalometric radiographs. Low-resolution cephalograms and subjects with craniofacial syndromes, trauma, cleft palate, and lip, previous orthognathic surgeries were excluded from the study. We examined lateral cephalometric radiographies of these patients. All the cephalograms of this study were taken using the PLANMECA device with the exposure of 66 kVp and 10 mA in the same cephalostat. Finally, 153 patients were included in this study divided into two groups: 80 patients (69 males and 11 females) had the long face development pattern while 73 patients (26 males and 47 females) had a normal face growth pattern as our control group. People with skeletal open bite were identified by considering the basal angle (larger than 27°).

**Figure 1:**
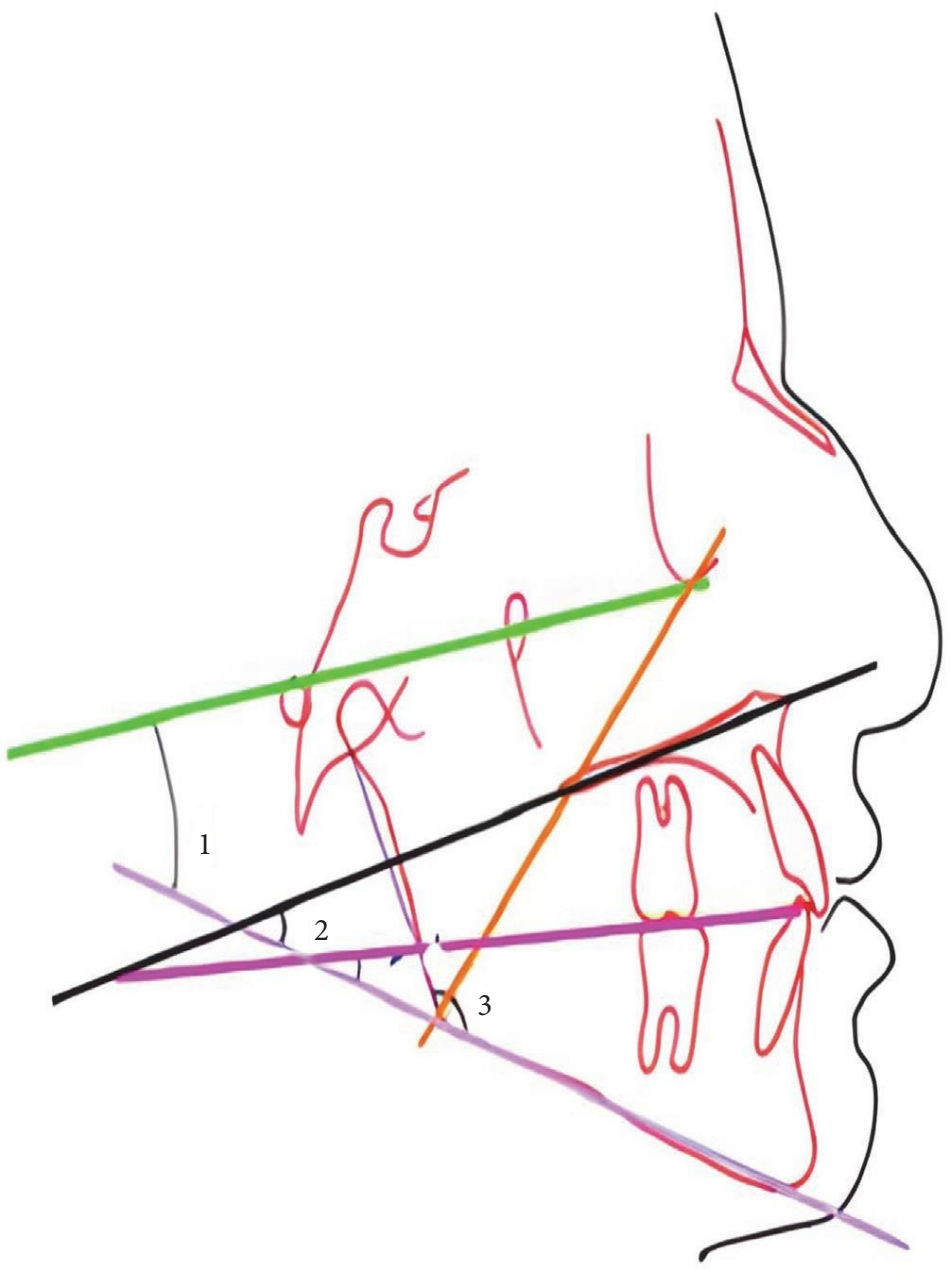
(1) FMA angle: the angle between the Frankfort plane and the mandibular plane; (2) basal angle: the angle between the mandibular plane and the palatal plane; (3) gonial angle: the angle between Ar (articular), Go (gonion), and Me (menton).

People's graphs were analyzed using the Romexis software, considering the three basal, gonial, and FMA angles (Figure 1), to determine the pattern of facial growth according to the following Table 1 [33]:

In this study, the patient whose three angles showed increased vertical growth range was considered as a patient with a long face to avoid entrance of borderline patients.

Also, to determine the dimensions of sella turcica, the location of four landmarks was determined on all cephalograms as follows [34]:

- TS (tuberculum sellae): anterior point of the contour of the sella turcica
- DS (dorsum sellae): the farthest point on the posterior wall of sella turcica
- Sellae floor: the deepest point on the base of the pituitary fossa
- Posterior clinoid: the most anterior point of the posterior clinoid process.

**Figure 2:**
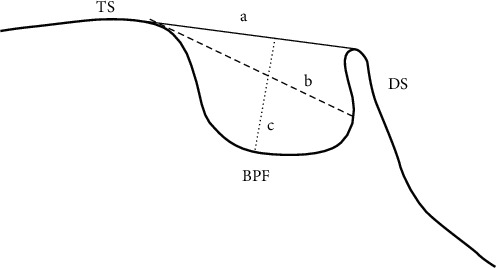
(a) Length of the sella turcica, (b) diameter of the sella turcica, and (c) depth of the sella turcica; base of the pituitary fossa (BPF).

The measurements were performed by a trained person twice, taking into account the landmarks defined according to the method provided by Silverman et al. [1]:

- Length of sella turcica: the linear distance from the TS to the tip of the posterior clinoid
- Depth of sella turcica: the vertical distance from the point where the length of sella is measured to the deepest point on the floor of sella turcica
- Anteroposterior diameter of sella turcica: the linear distance from the TS to the farthest point on the medial surface of the posterior wall of sella turcica (Figure 2).

**Figure 3:**
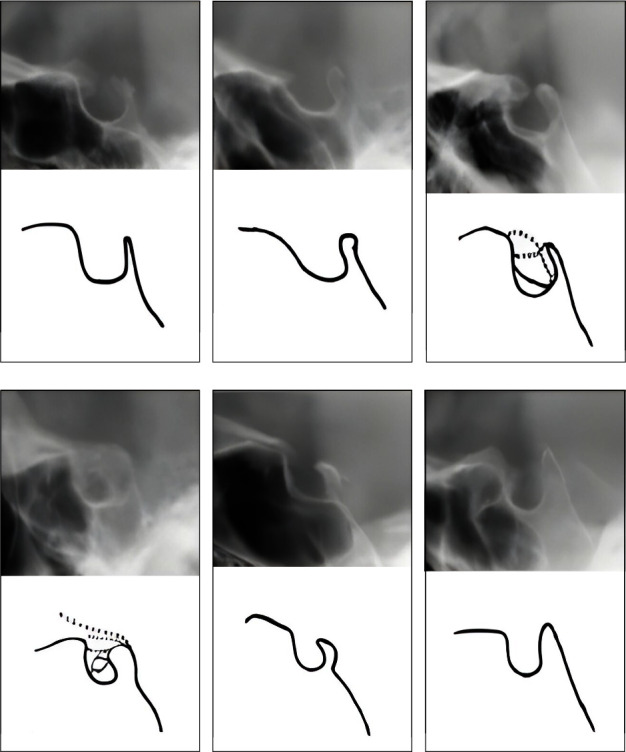
(a) Normal, (b) anterior oblique wall, (c) double floor contour, (d) sella turcica bridging, (e) irregularity in the posterior part of DS, and (f) the pyramidal shape of DS.

Another characteristic investigated in this study is the morphology of sella turcica, which according to the definition provided by Axelsson et al. [7], has six variations in shape, including: normal, anterior oblique wall, sella turcica bridging (STB), double floor contour, irregularity in the posterior part of DS, and the pyramidal shape of DS (Figure 3(a)–3(f)).

**Figure 4:**
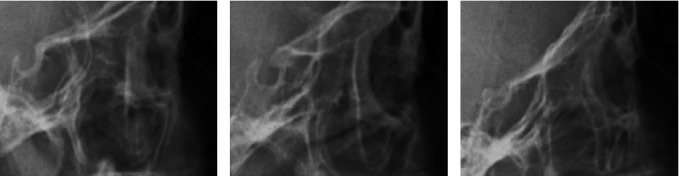
(a) Without bridging, (b) partial bridging, and (c) complete bridging.

STB has been classified according to the standard index provided by Leonardi et al. [6] based on the anteroposterior length and diameter of sella:

- Group I (without bridging): when the length of sella turcica is larger than ¾ of its diameter.
- Group II (partial bridging): when the length of sella turcica is equal to or smaller than ¾ of its diameter.
- Group III (complete bridging): when there is a visible contact between TS and DS in the radiography (Figure 4(a)–4(c)).

Then the above information was written in the checklist for each person, and finally the existence of a relationship between the dimensions and morphology of sella turcica and the long-face growth pattern was investigated.

The formula for comparing two proportions was used to determine the sample size as follows:

(1) $$\begin{array}{c} n={\left(Z_{1-\alpha /2}+Z_{1-\beta }\right)}^{2}\;\left[P_{1}\left(1-P_{1}\right)+P_{2}\left(1-P_{2}\right)\right]/{\left(P_{1}-P_{2}\right)}^{2}. \end{array}$$

P1 (exposure ratio in the case group) = 54.8, P2 (exposure ratio in the second group) = 51.4, *α* (type I error) = this value is 1.96 for an *α* of 0.05, *β* (type II error) = this value is 0.84 for a *β* of 0.20.

Logistic regression was used to investigate the relationship between the variables and to control confounding factors. univariate (crude) and multivariate (adjusted) logistic regression analyses were performed to determine the odds ratios (ORs) and 95% confidence intervals (CIs) for the relation of the length, depth, and anteroposterior diameter of the sella turcica with the occurrence of long faces in study participants. Variables with a *p* value below 0.05 in bivariate analysis were included in the regression models as potential confounders.

## 3. Results

Of the 153 people studied, 37 were men and 116 were women, with an average age of 16.83 years and a standard deviation of 5.11 years, of which 80 were classified as a long faced (11 men (13.75%) and 69 women (86.25%)) and 73 as normal (26 men (35.62%) and 47 women (64.38%)), where there was a significant difference between the two groups in terms of gender (*p* = 0.002). In terms of age, the average age of the long-faced group was 16.53 ± 4.73 while the normal group was 17.16 ± 5.51. Statistically, there was no age difference between the two groups (*p* = 0.44). Total 90 patients were classified as having skeletal open bites while 56 patients had normal skeletal bites.

As previously mentioned, gender was included in the regression models as a potential confounder due to a *p* value below 0.05 in multivariate analysis.

Crude and adjusted statistical models were used to investigate the relationship between the length, depth, and anteroposterior diameter of sella turcica and the occurrence of a long face. In the univariable or crude model, an increase in the length of the sella turcica for one unit decreased the chance of having a long face. However, in the adjusted model with bridging included, there was no significant relationship between length and the occurrence of a long face. In the crude model, an increase in the depth of the sella turcica was associated with with a greater chance of developing a long face. However, in the adjusted model, with the bridging factor included, there was not a significant relationship between depth and long face. There was not a significant relationship between the anteroposterior diameter of the sella turcica and the long face.

There was a significant relationship between the pyramidal shape and irregularity in the posterior part of the DS and the occurrence of a long face (Table 2).

The chance of developing a long face in people with partial and complete bridging was 8.38 and 1.92 times, respectively (*p* < 0.05). By controlling the confounding effect of gender, it was revealed that the chance of developing a long face in women is 3.8 times larger than in men. Also, by controlling the confounding effect of age, it was found that the chance of developing a long face decreases with age, but the relationship was not significant (*p* = 0.111) (Table 3).

In the single-variable model, an increase in the length of the sella turcica for one unit decreased the chance of developing an open bite. In the adjusted model, with the bridging included, a significant relationship did not exist between the length, depth, or anteroposterior diameter of sella turcica and the occurrence of an open bite (Table 4).

The ratio of the chance of developing an open bite in people with a pyramidal sella shape to people with a normal sella shape was 0.33 (Table 5).

The chance of developing an open bite in people with partial and complete bridging was 8.57 and 1.82 times larger than in people without bridging, respectively. Also, after controlling the confounding effect of sex, it was revealed that the chance of developing an open bite in women is 1.8 times larger than in men (Table 6).

Also, the length of sella turcica increased with age, but the relationship was not significant. Furthermore, the depth and the anteroposterior diameter of the sella turcica significantly increased with age.

The average length of the sella turcica in men was significantly larger than in women. The depth and anteroposterior diameter of the sella turcica in women were larger than in men, but this relationship was not significant.

A significant relationship was not found between the different shapes of sella turcica and sex (*p* = 0.107). The investigation of the relationship between the different shapes of sella turcica and age did not show a significant relationship (*p* = 0.86).

Based on the results shown in Table 7, there was no significant relationship between sex and the different types of bridging.

## 4. Discussion

The dimensions and morphology of sella turcica have been studied several times. Sella can be observed clearly in lateral cephalograms, and it has an important role in orthodontic diagnosis. Different studies have already found an association between normal and abnormal conditions and sella turcica morphology [35, 36].

The aim of the present study was to investigate the relationship between the dimensions and morphology of sella turcica, the long face, and the vertical growth patterns. The results of the present study showed that the occurrence of the long face abnormality has a negative relationship with the length of the sella turcica, a direct relationship with the depth of the sella turcica, and no relationship with the anteroposterior diameter of the sella turcica. Unlike the results of the present study, Alkofid [36] conducted a study to investigate the relationship between the shape and size of the sella turcica and skeletal classifications in lateral cephalometric images in Saudi patients and did not observe any obvious statistical difference in the length and depth of the sella turcica between Class 2 and 3 malocclusions, while there was a statistically significant difference in the diameter of the sella turcica between the different types of malocclusions. This difference is probably due to the investigation of the effect of the dimensions of the skull on two different planes of the skull. In Alkofid's [36] study, the effect of the dimensions of the skull on malocclusion in the anterior–posterior dimension was investigated, while the present study investigated malocclusion in the vertical dimension. Moreover, Tepedino et al. [37] also compared sagittal craniofacial patterns in patients ranging from 9 to 13 years old in terms of the shape and dimensions of the sella turcica. They suggested that the shape of the sella turcica in Class 1 patients is significantly different from that in Class 2 subjects, while the sella length or depth seems to be similar in all angle classes. They also reported that 13-year-old subjects have a larger sella than 9- to 10-year-old patients without any change in sella morphology [37]. This study shows that age may be an important factor affecting growth patterns as well as the shape and dimensions of sella turcica.

Recently, it has been proposed that STB is more frequent than thought. It is estimated that one in four subjects shows bridging. So it is expected that STB would enter anatomical and embryology textbooks while the world is looking for clear clinical implications and usage [38].

In the present study, the chance of developing a long face in people with partial and complete bridging was 8.37 and 1.92 times larger than in people without bridging, respectively. In line with the results of the present study, Buyuk et al. [5] concluded that there is a significant difference in the palatal plane-to-SN angle and the anterior height of the face in people with a normal shape of the sella turcica and people with partial or complete bridging of the sella. However, unlike the results of the present study, in Buyuk's study [5], there was no significant difference in the effect of bridging on cephalometric indices between people with partial bridging or complete bridging.

In the present study, after controlling for the confounding effect of sex, it was revealed that the chance of developing a long face in women is 3.8 times greater than in men. Unlike the results of the present study, Gupta et al. [39] investigated the prevalence of long faces among 100 adult patients (50 women and 50 men) in India and concluded that there is a slight difference in the prevalence of long faces in men compared to women. This difference is probably due to the high number of women compared to men in the present study.

It was also found that the chance of developing an open bite in people with partial and complete bridging is 57.8 and 82.1 times larger than in people without bridging, respectively. Also, after controlling for the confounding effect of sex, it was found that the chance of developing an open bite in women is 1.8 times greater than in men. Furthermore, in the adjusted model, an increase in the length of the sella turcica decreases the chance of developing an open bite. In the adjusted model, in which bridging is included, there was no significant relationship between the length of the sella turcica and the occurrence of a an open bite, which shows the significant effect of bridging on an open bite. In both the crude and the adjusted models, there was no significant relationship between the anteroposterior diameter or depth of the sella turcica and the occurrence of an open bite. Another finding of the present study was a negative relationship between length and a direct relationship between depth on the one hand and the long face abnormality on the other hand. Similar to the occurrence of an open bite, the anteroposterior diameter has no effect on the chance of developing a long face. In a study investigating the relationship between the shape and size of the sella turcica and skeletal classification, Valizadeh et al. [40] concluded that the length of the sella turcica has a significant effect on jaw relationships. However, unlike the results of the present study, which point to the effect of depth on the occurrence of long faces, Valizadeh et al. [40] did not find any effect of depth on jaw relationships, which could be due to the investigation of the effect of the dimensions of the skull in different planes of the skull in the present study.

This study had a retrospective nature with no 3D data, a low-target population with relatively many women, and patients with a wide age range. Designing a prospective study can make it possible to examine the growth rate in different dimensions, along with the changes in the dimensions of sella turcica over time. Moreover, the anteroposterior relationship between jaws has not been considered in this study, which is suggested to be evaluated in future studies. It is important to note that 3D data can affect the final results of a study, as the normal values for 3D data may differ from those of 2D data, leading to misses in long-face patients. It has been shown that the 3D method may reduce the chance of underestimating the angular measurements on the lateral landmarks as compared to 2D values, especially measurements not lying on the midsagittal plane, as in our recent study [41]. In addition, in recent studies, it seems that gender may have an effect on craniofacial measurements [42] so it is recommended to increase the number of men compared to the women in future studies for a better investigation. We also recommend more than three cephalometric angles be used for diagnosing vertical growth patterns in the future.

## 5. Conclusions

Bridging, length, and depth of the sella turcica can be associated with the occurrence of a long face. An open bite can also be associated with sella bridging as well as its length. The diameter and depth of the sella increase with age, while this is not significant for length.

## Figures and Tables

**Table 1:** Data analysis table for determining facial growth patterns.

Angle	Normal (°)	Long face (°)	Short face (°)
Frankfort-mandibular plane angle (FMA)	25 ± 2	Greater than 27	Smaller than 23
Gonial (Ar-Go-Me)	130 ± 7	Greater than 137	Smaller than 123
The angle between the palatal plane and the mandibular plane (basal angle)	25 ± 2	Greater than 27	Smaller than 23

**Table 2:** Investigating the relationship between the different shapes of sella turcica with long face.

Different shapes of sella turcica	Long face (%)	Normal face (%)	Total within-group (%)	Odds ratio	*p* Value
Normal	68.8	45.2	57.5	1	–
Sella turcica with anterior oblique wall	7.5	8.2	7.8	0.6	0.408
Double floor contour	6.3	6.8	6.5	0.6	0.446
Pyramidal shape	7.5	17.8	12.4	0.28	0.018
Irregularity in the posterior part of DS	10.0	21.9	15.7	0.3	0.013

**Table 3:** Investigating the relationship between sella turcica bridging and the occurrence of long face and the effects age and sex as dependent variables.

Different types of bridging	The crude model		The adjusted model^1,2^			
	Odds ratio (confidence interval)	*p* Value	Odds ratio (confidence interval)^1^	*p* Value	Odds ratio (confidence interval)^2^	*p* Value
Without bridging	1	–	1	–	1	–
Partial	8.37 (3.98–17.57)	0.00	8.61 (3.97–18.6)	0.00	9.61 (4.3–21.5)	0.00
Complete	1.92 (0.3–12.5)	0.50	1.41 (0.21–9.2)	0.72	2.37 (0.31–17.93)	0.40

The adjusted model: investigating the relationship between sella bridging and the occurrence of long face ^1^after controlling the confounding effect of sex, ^2^after controlling the confounding effect of age.

**Table 4:** Investigating the relationship among length, depth, and the anteroposterior diameter of sella turcica with the occurrence of open bite.

Variable	The crude model		The adjusted model^1^	
	Odds ratio (confidence interval)	*p* Value	Odds ratio (confidence interval)	*p* Value
Length	0.84 (0.74–0.98)	0.029	0.94 (0.74–1.12)	0.602
Depth	1.22 (0.92–1.5)	0.092	1.1 (0.83–1.3)	0.504
APT	1.14 (0.9–1.35)	0.201	1.01 (0.77–1.2)	0.892

^1^The adjusted model: the relationship among length, depth, and the anteroposterior diameter of sella turcica with the occurrence of open bite with bridging being included.

**Table 5:** Investigating the relationship between the different shapes of sella and open bite (*n* = 146).

Different shapes of sella turcica	Odds ratio	*p*-Value
Normal	1	–
Sella turcica with anterior oblique wall	0.90	0.87
Double floor contour	0.67	0.56
Pyramidal shape	0.33	0.032
Irregularity in the posterior part of DS	0.41	0.071

**Table 6:** Investigating the relationship between sella bridging and the occurrence of open bite after controlling the confounding effect of sex (*n* = 146).

Different types of bridging	The crude model		The adjusted model^1^	
	Odds ratio (confidence interval)	*p* Value	Odds ratio (confidence interval)	*p* Value
Without bridging	1	–	1	–
Partial	8.57 (3.58–15.94)	0.00	8.30 (3.47–15.6)	0.00
Complete	1.82 (0.3–14.7)	0.55	1.55 (0.31–13.44)	0.67

^1^The adjusted model: investigating the relationship between sella bridging and the occurrence of open bite after controlling the confounding effect of sex.

**Table 7:** The prevalence of different types bridging (without bridging, partial, and complete) by sex (*n* = 153).

Sex	Without bridging (%)	Partial (%)	Complete (%)	Total (%)
Male	30.3	20.7	0.00	24.2
Female	69.7	79.3	100	75.8
Total	100	100	100	100

## Data Availability

The underlying data of this study are available by contacting the corresponding author on reasonable request.
